# Association Between Periodontitis and Cancer: A Perspective Review of Mechanisms and Clinical Evidence

**DOI:** 10.3390/jcm14176334

**Published:** 2025-09-08

**Authors:** Marco Bonilla, Irene Peñalver, María José Mesa-López, Francisco Mesa

**Affiliations:** 1 Department of Periodontics, School of Dentistry, University of Granada, 18071 Granada, Spain; marcobonilla000@gmail.com; 2 School of Dentistry, University of Granada, 18071 Granada, Spain; penalverirene@correo.ugr.es; 3 Department of Digestive Diseases, Virgen de la Arrixaca University Hospital, 30120 Murcia, Spain; mari_ml_2@hotmail.com

**Keywords:** periodontitis, cancer, *Fusobacterium nucleatum*, cancer progression

## Abstract

Chronic periodontitis has emerged as a potential modifiable risk factor for several tumors, yet its role remains underexplored beyond epidemiological associations. This perspective review examines the immunological and molecular interplay between periodontitis and various cancers—including prostate, colorectal, oral squamous cell carcinoma, and oral potentially malignant disorders—highlighting shared inflammatory mediators and immune dysregulation. Special attention is given to immune cell profiles, cytokine expression, dysbiosis, and common miRNA signatures. Recent evidence suggests that periodontitis may act not only as a co-factor in tumor development but also, in some contexts, as a marker of therapeutic response, particularly in patients undergoing immune checkpoint inhibitor therapy. In our view, future research should prioritize mechanistic studies to define common immune–inflammatory pathways and clarify whether periodontitis functions as a field cancerization process or as a facilitator of malignant transformation in already compromised tissues. The relationship between cancer and periodontitis underscores the need to integrate oral health into oncologic care and immunotherapy management.

## 1. Introduction

For decades, periodontitis was regarded as a localized condition, confined to the periodontium and primarily associated with tooth loss [1]. However, with the emergence of periodontal medicine—a term describing the systemic impact of periodontitis—it is now recognized that this disease influences more than 50 systemic conditions, including cardiovascular, metabolic, and neurodegenerative disorders [2]. This reconceptualization has opened an emerging field of research linking oral dysbiosis to the development and progression of various types of cancer [3].

Among the periodontal pathobionts implicated in the oral dysbiosis–cancer axis, *Fusobacterium nucleatum* (*F. nucleatum*) has garnered particular attention. This anaerobic microorganism, commonly found in dysbiotic oral microbiota, has been identified at high concentrations in tumor tissues of the colon, esophagus, and oral cavity and is proposed to facilitate both tumorigenesis and resistance to chemotherapeutic treatment [4]. Its ability to adhere to epithelial cells, invade tissues, disrupt local immunity [5], and promote macrophage polarization toward an immunosuppressive phenotype makes it a key player in the link between periodontal inflammation and tumor progression [6]. Furthermore, *F. nucleatum* has been implicated in the activation of signaling pathways such as NF-κB, STAT3, and β-catenin as well as in the induction of molecules like CXCL8, IL-6, and MMPs, all of which play recognized roles in carcinogenesis [7,8].

Recent evidence suggests that chronic inflammation plays a pivotal role across all stages of cancer development, from initiation to metastasis, through mechanisms such as suppression of antitumor immunity and the production of inflammatory and genotoxic mediators [9], thereby establishing a microenvironment conducive to tumor transformation.

Although multiple studies and systematic reviews have described a connection between certain periodontal pathobionts and carcinogenesis, there is still no critical synthesis specifically addressing the role of periodontitis as a clinical entity. We hypothesize that periodontitis—as a form of low-grade chronic inflammation—may act as a systemic modulator of oncologic risk.

Therefore, the aim of this perspective review is to analyze, for the first time, the available literature on the association between periodontitis and various types of cancer, with particular emphasis on the immunological, microbial, and molecular mechanisms that may unravel this pathogenic link.

## 2. Materials and Methods

### 2.1. Search Strategy

A comprehensive literature search was performed in the PubMed (MedLine), Scopus, Web of Science, and Google Scholar electronic databases. To reduce selection and publication bias, grey literature sources, including OpenGrey, OpenThesis, LILACS, and TESEO, were also searched. The search was independently conducted by two reviewers (M.J.M.-L. and I.P.-F.) between 6 July and 15 July 2025.

The search strategy combined controlled vocabulary terms (e.g., MeSH) and free-text keywords, using Boolean operators (“AND”, “OR”, “NOT”) to refine the queries. The final search terms included combinations of (“periodontitis” OR “periodontal disease” OR “gum disease” OR “fusobacterium nucleatum” OR “porphyromonas gingivalis” OR “aggregatibacter actinomycetemcomitans” OR “periodontal bacteria”) AND (“cancer” OR “carcinoma” OR “neoplasm” OR “tumor” OR “malignancy”) AND (“mechanism” OR “pathway” OR “molecular mechanism” OR “dysplasia” OR “anoikis” OR “metastasis” OR “inflammatory environment” OR “carcinoembryonic antigen” OR “CEA” OR “cell death” OR “immune response”).

### 2.2. Eligibility Criteria

#### 2.2.1. Research Question and PECOS Strategy

The research question for this perspective review is the following: “What immunological, inflammatory, or molecular mechanisms link periodontitis to cancer or precancerous lesions in human populations?”

The PECOS strategy applied is as follows:

P (Population): Human subjects with any type of cancer or precancerous lesions.

E (Exposure): Periodontitis clinically diagnosed or assessed through recognized clinical parameters (e.g., probing depth, clinical attachment loss).

C (Comparison): Individuals with healthy periodontal status or with differing degrees of periodontal disease severity.

O (Outcomes): Immunological, inflammatory, or molecular mechanisms associated with cancer initiation, progression, or tumor microenvironment modulation.

S (Study Design): Clinical observational studies.

#### 2.2.2. Inclusion Criteria

Clinical studies: case–control, cohort, and cross-sectional designs;

Ex vivo human studies: analyses based on biopsies, gingival crevicular fluid (GCF), or saliva samples;

Studies evaluating immunological, inflammatory, or molecular mechanisms linking periodontitis to cancer or precancerous lesions;

All cancer types are eligible, provided that the study explores a mechanistic or immunological association with periodontitis;

Studies published between January 2023 and June 2025;

No language restrictions will be applied.

#### 2.2.3. Exclusion Criteria

In vitro or animal studies;

Purely descriptive epidemiological studies;

Studies focusing on gingivitis or the gut microbiota;

Studies analyzing isolated periodontal pathogens without clinical context.

### 2.3. Selection of Studies

Two independent, blinded reviewers (M.J.M.-L and I.P.-F.) applied the predefined eligibility criteria to all retrieved records. The study selection process was performed in two consecutive phases: Phase I involved the screening of titles and abstracts, and Phase II consisted of a full-text evaluation of records deemed potentially eligible in the previous phase. Discrepancies were resolved through discussion and consensus, with the involvement of a third author (F.M.) when necessary. Prior to the formal screening, both reviewers were trained and calibrated by conducting pilot screening rounds of 100 randomly selected records. This process yielded an inter-reviewer agreement rate of 90%. Inter-rater reliability was calculated using Cohen’s kappa (κ > 0.800), indicating almost perfect agreement. Articles not published in English were screened and evaluated by the reviewers in their original language whenever possible. If necessary, professional translation services were used to ensure accurate evaluation and data extraction.

### 2.4. Data Extraction

Data extraction was performed independently by the two reviewers using a standardized spreadsheet created in Microsoft Excel. The extracted variables included author and year, type of study, sample size, biological sample analyzed, type of cancer, and major findings. Extracted data were then synthesized qualitatively, summarizing patterns and key findings across studies to identify common immunological and microbiological mechanisms linking periodontitis with cancer. No quantitative meta-analysis was performed due to the heterogeneity of study designs and outcomes.

## 3. Results

### 3.1. Results of the Literature Search

A total of 1599 articles were identified: 461 from PubMed/MEDLINE, 612 from Web of Science, and 526 from Scopus. After removing duplicates, non-original papers, and records falling outside the scope of this perspective paper, 388 records were screened by title and abstract. Of these, 373 were excluded for not meeting the inclusion criteria. Ultimately, seven studies were included in the final synthesis [10,11,12,13,14,15,16].

### 3.2. Study Characteristics

Table 1 provides a summary of the main characteristics of the included studies. This review comprised seven clinical ex vivo studies, encompassing a total of 1762 patients with cancer or precancerous lesions. The selection included one study on colorectal cancer (CRC) [10], three studies on oral squamous cell carcinoma (OSCC) [11,12,15], one study on prostate cancer (PC) [13], one study involving various cancer types [14], and one study on oral lichen planus (OLP) [16].

In the PC study, a higher prevalence (54.6% vs. 41.4%) and greater severity of periodontitis were reported in patients with PC than in controls [13]. These patients also exhibited worse periodontal conditions, including a lower number of teeth, increased gingival bleeding (BOP), a larger periodontal inflamed surface area (PISA), and a greater percentage of sites with a probing pocket depth (PPD) of 5–6 mm and ≥7 mm. The presence of periodontitis was significantly associated with the occurrence of PC, although the increase in prostate-specific antigen levels in relation to periodontal severity did not reach statistical significance.

Regarding CRC, the prevalence of periodontitis was significantly higher among patients with cancer (69.5%) than among the general population (40%) [10]. Additionally, both baseline and peak carcinoembryonic antigen (CEA) levels were higher in the periodontitis group, whereas no significant differences were found in tumor histopathological variables. These findings suggest a potential contribution of periodontal inflammation to tumor susceptibility, without a direct impact on CRC severity.

Oral squamous cell carcinoma (OSCC) and potentially malignant lesions showed no significant differences in IL-23R expression or macrophage markers (CD68, CD11c, CD163) between healthy and periodontitis-affected tissues [11]. However, increased levels of these markers were observed in oral leukoplakia and oral lichen planus (OLP). These lesions exhibited a higher proportion of M2 macrophages and increased cell density following anti-PD1 immunotherapy. More than 50% of IL-23R-positive cells co-expressed myeloid markers, suggesting an active role of the IL-23/macrophage axis in the tumor microenvironment.

Huang et al. [12] reported that clinical periodontal parameters—clinical attachment loss (CAL), BOP, and PPD—were significantly higher in patients with OSCC and periodontitis than in both healthy controls and patients with periodontitis alone. A reduction in microbial diversity and TCRβ repertoire was noted, with oligoclonal expansion of specific clones in tumor tissue.

Reddy et al. [15] observed a significantly higher incidence of periodontitis in patients with OSCC (63.9% vs. 32.4%), with a predominance of advanced periodontitis (stage 4). Risk factors associated with oral cancer included smoking, alcohol consumption, low educational level, and male gender. Notably, 7.9% of OSCC cases had severe periodontitis as the sole identified risk factor.

In patients treated with immune checkpoint inhibitors (ICIs), Ma KS et al. [14] observed a higher incidence of periodontitis than in untreated patients (55.3 vs. 25.8 cases per 100 patient-years). Immune-related periodontitis was associated with an increased risk of cutaneous adverse events and improved overall survival. Predictive factors included younger age, prior radiotherapy, and previous immune-related adverse events.

Finally, in patients with OLP, coexistence with periodontitis increased the risk of developing oral precancerous lesions [16]. Elevated levels of MMP-1 and MMP-9 were also detected, suggesting a potential mechanism for tissue degeneration and carcinogenesis.

## 4. Discussion

The results of this perspective review demonstrate an association between periodontitis and various types of cancer, including PC, CRC, OSCC, and potentially malignant lesions such as OLP. In the included studies, periodontitis was consistently linked to a higher prevalence and severity of these neoplasms, as well as to immunological, inflammatory, and molecular alterations that may serve as underlying mechanisms. Specifically, patients with cancer exhibited worse periodontal parameters, increased expression of inflammatory mediators, overexpression of microRNAs associated with tumor progression, and altered immune cell infiltration in affected tissues.

### 4.1. Periodontitis and PC

The interaction between periodontitis and PC appears to be mediated by shared immunoinflammatory and molecular mechanisms, beyond a mere epidemiological association. PC is the second most common cancer in men and the fifth leading cause of cancer-related mortality worldwide [17]. Periodontitis has been identified as a potential risk factor for PC, supported by two recent meta-analyses reporting an increased risk of PC in patients with periodontitis of 1.17 [18] and 1.4 times [19]. This link may be explained by chronic inflammation, which promotes carcinogenesis through alterations in the tissue microenvironment and modulation of immunity [20].

The study by Vitor et al. [13] reported a higher prevalence and greater severity of periodontitis in patients with PC than in controls, along with worse periodontal parameters such as fewer teeth, increased BOP, elevated inflammatory indices, and a higher percentage of sites with PPD ≥ 5 mm. A significant association between periodontitis and the presence of PC was observed. At the molecular level, 12 differentially expressed miRNAs common to both periodontitis and PC were identified [21], 8 of which exhibited similar patterns of upregulation or downregulation, notably hsa-mir-148a-3p, hsa-mir-148b-5p, and hsa-mir-623. These miRNAs are involved in processes related to inflammation, cell fate, and tumor progression [22]; for instance, the miR-148/152 family regulates immune and hematopoietic function [23], while hsa-mir-623 is implicated in the oral epithelial cell response to *Porphyromonas gingivalis* (*P. gingivalis*) [24]. Additionally, transcription factors such as TP53, CREB1, and DNMT1 stand out [21], consolidating a molecular network that could link chronic periodontal inflammation with prostatic carcinogenesis.

### 4.2. Periodontitis and CRC

In the case of CRC, one study proposes that periodontitis may act as field cancerization [10] by inducing an inflammatory microenvironment that promotes the release of CEA. In this context, patients with periodontitis exhibited significantly higher CEA levels than those without periodontitis, suggesting a possible involvement in the early stages of tumor development. However, the findings suggest that periodontitis does not directly influence the severity or progression of colorectal cancer. Elevated CEA levels are associated with increased aggressiveness, lymphovascular invasion, and reduced survival. Furthermore, CEA can interfere with pro-apoptotic signaling by inhibiting anoikis, thereby facilitating metastasis [25]. These results are consistent with those reported by Luo et al., who found that periodontitis increases the risk of colon cancer by up to 2.3-fold and rectal cancer by 2.7-fold, remaining a significant risk factor even after adjustment for confounding variables, and is particularly associated with a higher risk of distant metastases in severe cases [26].

Another common mechanism between periodontitis and CRC is IL-6 trans-signaling. Periodontitis acts as a chronic source of IL-6, which, besides promoting periodontal tissue destruction [27], has been linked to the development and progression of CRC. In this context, IL-6 trans-signaling stimulates tumor cell proliferation and inhibits apoptosis through activation of gp130, JAKs, and STAT3 in tumor cells [28].

**Figure 1:**
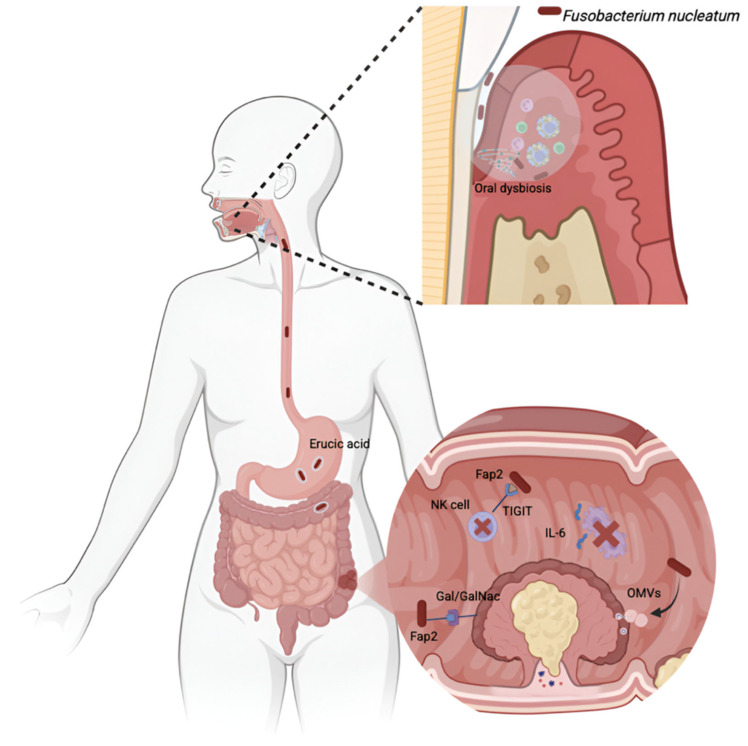
*Fusobacterium nucleatum* (*F. nucleatum*) as a microbial bridge between periodontitis and colorectal cancer (CRC). Chronic periodontitis induces oral dysbiosis and facilitates the dissemination of *F. nucleatum* to the colon. This bacterium survives the acidic gastric environment through the accumulation of erucic acid in its membrane, which provides acid resistance and enables gastrointestinal translocation. In the tumor microenvironment, *F. nucleatum* colonizes CRC tissue via the adhesin Fap2, which binds to the Gal/GalNAc receptor, and suppresses antitumor immunity by interacting with the TIGIT receptor on NK and T cells. Additionally, it induces the secretion of interleukins (IL-8, IL-6, CXCL1), outer membrane vesicles (OMVs), and overexpression of carcinoembryonic antigen, contributing to inflammation, immune evasion, and metastatic potential. Notably, IL-6 trans-signaling inhibits apoptosis, further promoting tumor survival and progression.

The importance of periodontopathogens, especially *F. nucleatum*, should be highlighted, as *F. nucleatum* emerges as a key player in CRC due to its high abundance in tumor tissue, association with poor prognosis, and involvement in chemotherapy-resistance mechanisms (Figure 1). *F. nucleatum* promotes tumor progression by activating the TLR4/NF-κB pathway, directly adhering to tumor cells, increasing IL-8 production, and inhibiting ferroptosis [29]. Among its most relevant virulence factors is the adhesin FadA, which binds to E-cadherin on CRC cells, activates the β-catenin pathway, and stimulates the expression of oncogenes such as Myc and Cyclin D1, as well as inducing a proinflammatory response through IL-6, IL-8, and IL-18 [30]. Complementarily, the surface protein Fap2 contributes to tumor tropism by recognizing Gal/GalNAc disaccharides, highly expressed in neoplastic tissues, facilitating tumor colonization. Additionally, Fap2 interferes with the immune response by activating the TIGIT receptor on T and NK cells, reducing their cytotoxic capacity and fostering an immunosuppressive microenvironment [31].

Additionally, the ability of *F. nucleatum* to survive the gastric environment through erucic acid production and the expression of the FnFabM gene allows its translocation from the oral cavity to the intestine [32]. Its presence in the hypoxic tumor microenvironment favors infection, intracellular persistence, epigenetic reprogramming of tumor cells, and induction of cytokines such as CXCL1, reinforcing more aggressive phenotypes [33]. It has also been shown to induce microsatellite instability through suppression of MSH6 [34], facilitate liver metastasis via the miR-5692a/IL-8 axis [35], and modulate the tumor microenvironment through the outer membrane vesicles (OMVs) that induce immunosuppressive M2 macrophage polarization [36].

*P. gingivalis*, on the other hand, may also promote tumor progression by inducing a Th2 response and M2 macrophage-mediated immunosuppression [37]. Finally, both periodontitis and CRC are characterized by elevated levels of CD8+ cells, regulatory T cells, and PD-1+ T cells, which could suppress the antitumor immune response [38].

### 4.3. Periodontitis and OSCC

Concerning OSCC, the relationship with periodontitis remains controversial. Trumet et al. [11] found no significant differences in IL-23R expression or macrophage markers (CD68, CD11c, CD163) between healthy gingival tissue and periodontitis-affected tissue. However, an increase in these markers was observed in oral leukoplakia and OLP, especially following anti-PD-1 immunotherapy, with increased cellular density and infiltration of M2 macrophages. More than 50% of IL-23R-positive cells co-expressed myeloid markers, suggesting an immunomodulatory role of the IL-23/macrophage axis in the tumor microenvironment of oral precursor lesions.

On the other hand, Huang et al. [12] reported that clinical parameters were significantly worse in patients with OSCC and periodontitis. Additionally, a reduction in oral microbial diversity and TCRβ repertoire was observed, alongside oligoclonal expansion of specific clones in tumor tissue, indicating a locally altered immune response possibly influenced by chronic periodontal inflammation.

From an epidemiological perspective, Reddy et al. [15] reported a significantly higher prevalence of periodontitis in patients with OSCC (63.9% versus 32.4%), with a predominance of advanced stages (stage IV), and in 7.9% of cases, severe periodontitis was the only identifiable risk factor.

From a molecular standpoint, *P. gingivalis* promotes the proliferation and migration of oral tumor cells through gingipains and OMVs and induces chemotherapy resistance [39,40]. *F. nucleatum*, in turn, favors polarization of M2 macrophages, activates NF-κB, and decreases infiltration of cytotoxic T cells, creating an immunosuppressive environment that promotes tumor progression as part of the previously described mechanism [6]. Both bacteria have also been shown to induce IL-6 production [41], which appears to play a pivotal role in the tumor microenvironment of OSCC and other head and neck squamous cell carcinomas. Elevated serum and tissue levels of IL-6 have been consistently reported in patients with OSCC, particularly in advanced stages and in cases with lymph node metastases [42]. Furthermore, the coexistence of periodontitis and cancer seems to potentiate IL-6 production, potentially leading to increased regulatory T-cell infiltration and promoting tumor progression [43].

### 4.4. Immune-Related Periodontitis

Recent findings suggest a complex interaction between immunotherapy and periodontitis, indicating a bidirectional relationship. On the one hand, preexisting periodontitis has been proposed as a negative modulating factor since by inducing chronic systemic inflammation and oral and intestinal dysbiosis, it may reduce therapeutic efficacy and increase susceptibility to immune-related adverse events (irAEs), as indicated by the findings of Pai et al. [44]. On the other hand, recent studies such as that of Ma et al. [14] have shown that periodontitis can also arise as an irAE directly induced by ICIs, with a significantly higher incidence in patients treated with these agents. Interestingly, the same study observed that patients who developed periodontitis after initiating ICIs exhibited improved overall survival, suggesting that the induced periodontal inflammation might reflect heightened systemic immune activation with antitumor effects.

Taken together, these data point to a complex interplay in which periodontitis may act, depending on timing and context, both as a negative prognostic factor and as a marker of favorable response. This duality underscores the need to integrate periodontal assessment into the planning and follow-up of patients with cancer undergoing immunotherapy.

### 4.5. Periodontitis and OLP

Several recent studies have delved into the complex relationship between OLP and periodontitis, revealing a bidirectional interaction with significant clinical implications. Systematic reviews and meta-analyses conducted by Sanadi et al. [45] and Nunes et al. [46] confirm a significant association between these conditions, with a notable deterioration in periodontal parameters in patients with OLP. Specifically, increases in BOP, PPD, and CAL have been observed. Plaque accumulation was attributed to difficulties in maintaining adequate oral hygiene due to pain and inflammation caused by erosive or atrophic forms of OLP. At the immunoinflammatory level, an exacerbated response has been described, characterized by elevated levels of interleukins (IL-17, IL-23) and MMPs as well as alterations in the subgingival microbiota [16].

### 4.6. Future Directions

In our view, the immunological and microbiological interplay between periodontitis and cancer described in this perspective review highlights the crucial role of chronic periodontal inflammation in the development of various types of cancer. However, despite growing evidence, most studies remain observational or exploratory, and only seven articles included in this perspective review analyze an immunological link between these pathologies.

As authors, we believe future research should prioritize the identification of common inflammatory and molecular signatures between periodontitis and specific cancers—particularly focusing on immune checkpoints and myeloid responses. Considering the recent concept of a “field of cancerization” proposed for periodontitis [10], we consider it plausible that this disease acts as a facilitating inflammatory environment, sharing key immunopathological pathways with carcinogenesis. Future research should also investigate IL-6 as a key driver of cancer progression across multiple malignancies. Mechanistically, IL-6 promotes tumor growth, metastasis, angiogenesis, immune modulation, and therapy resistance through gp130-mediated signaling. It also acts synergistically with cofactors such as *Helicobacter pylori* infection, obesity, and ulcerative colitis [47]. These pathways represent promising targets for therapeutic intervention and may provide a mechanistic framework linking periodontitis with cancer development.

However, since no common mechanistic pathways were found between OLP—as a precancerous lesion—and periodontitis, this suggests that periodontitis does not act as a precancerous lesion per se. Rather, it appears to function as a promoting factor that facilitates malignant transformation in previously altered tissues through disruption and dysregulation of the local microenvironment.

Conversely, the onset of periodontitis following immunotherapy has been associated with improved prognosis and survival. We hypothesize that, given that immunotherapy promotes a more robust systemic immune activation, this may lead to inflammation in peripheral tissues (such as the periodontium) while simultaneously enhancing an effective antitumor response. This duality suggests the coexistence of two distinct periodontitis profiles in the oncological context: one linked to a dysfunctional immune system—the “classic” periodontitis—that favors carcinogenesis, and another representing a collateral manifestation of therapeutically beneficial immune activation.

Overall, integrating periodontal assessment into oncological practice could provide not only an opportunity to improve oral health but also a diagnostic window into the patient’s immunological status, thereby opening new avenues for personalized medicine. This review, however, presents several limitations. The small number of eligible studies and their heterogeneity—in terms of study design and cancer endpoints—limits the strength and generalizability of the findings. Moreover, the available evidence is observational or exploratory, often lacking standardized protocols for immunological and microbiological assessments, which may introduce variability and reporting bias. From a future perspective, it is essential to design and conduct randomized clinical trials to evaluate the impact of periodontal treatment on the survival of patients with cancer as well as on relapse incidence, clinical progression, and the modulation of relevant immunological and tumor biomarkers.

## 5. Conclusions

The evidence gathered in this review supports a pathogenic interaction between periodontitis and various types of cancer, mediated by shared immunoinflammatory pathways, dysbiosis, and common molecular mechanisms. This convergence suggests that periodontitis may not only increase cancer risk but also influence its clinical progression and therapeutic response, highlighting the need to integrate dental care into oncological management.

## Figures and Tables

**Table 1:** Summary of the main characteristics of the included studies. *CRC*, colorectal cancer; *CEA*, carcinoembryonic antigen; *OSCC*, oral squamous cell carcinoma; *PC*, prostate cancer; *PPD*, probing pocket depth; *BOP*, bleeding on probing; *CAL*, clinical attachment loss; *PISA*, periodontal inflamed surface area; *ICI*, immune checkpoint inhibitor; *irAEs*, immune-related adverse effects; *P. gingivalis*, *Porphyromonas gingivalis*; *OLP*, oral lichen planus.

Author/Date	Study Design	N	Sample	Cancer Type	Main Outcomes
Mesa-López et al., 2025 [10]	Cross-sectional	59 patients	Colonoscopy biopsy	CRC	In total, 69.5% of patients with CRC presented with periodontitis. A significant association was observed between CEA and periodontitis, likely due to the role of chronic inflammation. Periodontitis may create a favorable microenvironment for cancer development, acting like field cancerization, but does not appear to influence disease progression.
Trumet et al., 2025 [11]	Pilot	29 patients with cancer and periodontitis	Gingiva biopsy	OSCC	Periodontitis showed a trend toward increased macrophage infiltration, similar to precancerous lesions and OSCC, suggesting a potential shared inflammatory axis in oral carcinogenesis.
Huang et al., 2025 [12]	Cross-sectional	5 healthy, 5 moderate, 5 severe periodontitis with OSCC	Subgingival plaque and gingival/tumor tissue	OSCC	Microbial dysbiosis and reduced TCRβ repertoire diversity in patients with periodontitis and OSCC suggest a possible immuno-microbial pathway in oral carcinogenesis.
Vitor et al., 2025 [13]	Case–control	152 cases, 220 controls	Periodontal examination	PC	Periodontitis was significantly associated with PC. Patients with PC exhibited worse periodontal status (higher PPD, CAL, BOP, and PISA). The association was stronger in patients with lower Gleason grades. Chronic inflammation may mediate this link.
Ma et al., 2024 [14]	Cohort	868 treated with ICIs	Periodontal examination and irAEs	Various types of cancer	ICI treatment was associated with an increased risk of periodontitis. Interestingly, immune-related periodontitis was linked to improved overall and progression-free survival as well as concurrent immune-related cutaneous adverse events.
Reddy et al., 2024 [15]	Case–control	63 cases, 63 controls	Questionnaire	OSCC	Periodontitis severity was significantly associated with oral cancer risk. Chronic inflammation, shifts in the oral microbiome, and cofactors such as smoking, alcohol, and poor oral hygiene contribute. Advanced periodontitis may act as an independent risk factor even in the absence of smoking or alcohol. Proinflammatory cytokines (IL-1β, TNF-α), pathogens like *P. gingivalis*, and oncogenic viruses contribute to carcinogenesis.
Huang et al., 2023 [16]	Retrospective	293 patients	Biopsy	OLP	Periodontitis in patients with OLP is associated with a higher risk of precancerous lesions, possibly mediated by MMP-1/MMP-9, IL-17, and TNF-α. Additional risk is conferred by factors such as betel nut use, tobacco, and *Candida* infection.

## Data Availability

Not applicable.
